# Rosiglitazone ameliorates palmitic acid-induced cytotoxicity in TM4 Sertoli cells

**DOI:** 10.1186/s12958-018-0416-0

**Published:** 2018-10-17

**Authors:** Xie Ge, Peng Pan, Jun Jing, Xuechun Hu, Li Chen, Xuhua Qiu, Rujun Ma, Kadiliya Jueraitetibaike, Xuan Huang, Bing Yao

**Affiliations:** Center of Reproductive Medicine, Nanjing Jinling Hospital, Clinical School of Medical College, Nanjing University, Nanjing, 210002 Jiangsu China

**Keywords:** Rosiglitazone, Palmitic acid, Sertoli cells, Cytotoxicity

## Abstract

The Sertoli cell is the only somatic cell within the seminiferous tubules, and is vital for testis development and spermatogenesis. Rosiglitazone (RSG) is a member of the thiazolidinedione family and is a peroxisome proliferator-activated receptor-γ (PPARγ) agonist. It has been reported that RSG protects various types of cells from fatty acid-induced damage. However, whether RSG serves a protective role in Sertoli cells against palmitic acid (PA)-induced toxicity remains to be elucidated. Therefore, the aim of the present study was to investigate the effect of RSG on PA-induced cytotoxicity in Sertoli cells. MTT assay and Oil Red O staining revealed that RSG ameliorated the PA-induced decrease in TM4 cell viability, which was accompanied by an alleviation of PA-induced lipid accumulation in cells. In primary mouse Sertoli cells, RSG also showed similar protective effects against PA-induced lipotoxicity. Knockdown of PPARγ verified that RSG exerted its protective role in TM4 cells through a PPARγ-dependent pathway. To evaluate the mechanism underlying the protective role of RSG on PA-induced lipotoxicity, the present study analyzed the effects of RSG on PA uptake, and the expression of genes associated with both fatty acid oxidation and triglyceride synthesis. The results demonstrated that although RSG did not affect the endocytosis of PA, it significantly elevated the expression of carnitine palmitoyltransferase (CPT)-1A, a key enzyme involved in fatty acid oxidation, which indicated that the protective effect of RSG may have an important role in fatty acid oxidation. On the other hand, the expression of CPT1B was not affected by RSG. Moreover, the expression levels of diacylglycerol O-acyltransferase (DGAT)-1 and DGAT2, both of which encode enzymes catalyzing the synthesis of triglycerides, were not suppressed by RSG. The results indicated that RSG reduced PA-induced lipid accumulation by promoting fatty acid oxidation mediated by CPT1A. The effect of RSG in protecting cells from lipotoxicity was also found to be specific to Sertoli cells and hepatocytes, and not to other cell types that do not store excess lipid in large quantities, such as human umbilical vein endothelial cells. These findings provide insights into the cytoprotective effects of RSG on Sertoli cells and suggest that PPARγ activation may be a useful therapeutic method for the treatment of Sertoli cell dysfunction caused by dyslipidemia.

**Electronic supplementary material:**

The online version of this article (10.1186/s12958-018-0416-0) contains supplementary material, which is available to authorized users.

## Background

Sertoli cells, located in the basal compartment of seminiferous tubules, play an important role in testis development and spermatogenesis. They not only secrete functional proteins for the regulation of spermatogonia proliferation and differentiation, but also secrete hormones, such as inhibin B and anti-Mullerian hormone [1, 2]. Moreover, Sertoli cells form cell junctions between themselves or with germ cells, either to construct a blood-testis barrier to provide a separated microenvironment for spermatogenesis, or to bind with germ cells to regulate their development [3, 4]. In fact, the majority of nutrients required for spermatogenesis, including lactates and lipids, are provided by Sertoli cells [5]. Therefore, the number of Sertoli cells defines the population size of germ cells, which is essential for the maintenance of spermatogenesis and consequently, male fertility [6].

In recent years, due to the increase in obesity as well as the rising rates of male infertility, the relationship between obesity and male infertility has drawn an increasing level of public attention [7]. As obesity is usually accompanied by elevated fatty acid levels, especially saturated fatty acids such as palmitic acid (PA) [8], it is thought that increased levels of saturated fatty acids may be a risk factor for male infertility caused by obesity. PA is the most common type of saturated fatty acid in the plasma, and has been reported to be toxic to various types of cells, including pancreatic β-cells, hepatocytes and retinal ganglion cells [9–11]. PA is also the major saturated fatty acid in human spermatozoa; some previous studies have indicated that there may be a relationship between PA concentration in the spermatozoa and male infertility [12, 13]. An in vitro study also demonstrated the proapoptotic effect of PA on Leydig cells, which are located in the interstitial space of the testis [14]. Moreover, according to our previous study, PA decreased Sertoli cell viability by inducing apoptosis (unpublished data). Therefore, excess PA may be harmful to the testes and negatively affect spermatogenesis. Ameliorating the toxic effects of PA on testis cells, including Sertoli cells, may be an effective method to treat male infertility coupled with obesity.

Rosiglitazone (RSG) is a member of the thiazolidinedione class of drugs; it exerts anti-diabetic effects by activating peroxisome proliferator-activated receptor-γ (PPARγ). It has also been reported to have beneficial effects on lipid accumulation in the liver [15], and it previously ameliorated dyslipidemia in obese mice [16]. A number of studies have reported that RSG has protective roles in fatty acid-induced cell toxicity, including in pancreatic β-cells and skeletal muscle cells [17, 18]. In both of these types of cells, PA-induced apoptosis was ameliorated following treatment with RSG. Therefore, it is possible that RSG may protect Sertoli cells from PA-induced damage. To test this hypothesis, the present study investigated the effect of RSG on PA-induced cytotoxicity in Sertoli cells.

## Methods

### Materials

RSG was purchased from Aladdin Reagents (Shanghai, China), and was dissolved in dimethyl sulfoxide (DMSO; Sigma-Aldrich, Shanghai, China) to generate a 200 mM stock for subsequent use. PA was purchased from Sigma-Aldrich. For cell treatments, PA was dissolved in ethanol to create a 600 mM solution and then diluted with Dulbecco’s modified Eagle’s medium/Ham’s nutrient mixture F12 (DMEM/F12; Yuanye, Shanghai, China) containing 2% fatty acid free-bovine serum albumin (Yeasen, Shanghai, China) to a final concentration of 10 mM, which was used as a stock for further experimentation. 3-(4,5-Dimethyl-2-thiazolyl)-2,5-diphenyl-2-H-tetrazolium bromide (MTT) was purchased from Biosharp (Hefei, China), and dissolved in phosphate buffer saline (PBS) to produce a 5 mg/ml stock. Oil Red O (ORO) was purchased from Sigma-Aldrich, and dissolved in isopropanol to generate a 5 mg/ml stock, which was diluted before use with distilled water to produce a 3 mg/ml working solution. 4,4-Difluoro-5,7-dimethyl-4-bora-3a,4a-diaza-s-indacene-3-hexadecanoic acid (BODIPY FL C16) was purchased from Invitrogen (Thermo Fisher Scientific, Inc., Waltham, MA, USA) and dissolved in DMSO to create a 2 mM stock for subsequent use.

### TM4 cell culture

The TM4 cell line was purchased from iCell Bioscience, Inc. (Shanghai, China). The HepG2 [American Type Culture Collection (ATCC)® HB-8065™] and human umbilical vein endothelial cells (HUVECs; ATCC® PCS-100-010™) cell lines were purchased from ATCC (Manassas, VA, USA). TM4 cells and HUVECs were cultured in DMEM/F12 supplemented with 10% fetal bovine serum (FBS; Gibco; Thermo Fisher Scientific, Inc.) at 37 °C in 5% CO_2_. HepG2 cells were cultured in DMEM supplemented with 10% FBS at 37 °C in 5% CO_2_.

### Primary mouse Sertoli cell isolation and culture

Male ICR mice were purchased from Beijing Vital River Laboratory Animal Technology Co., Ltd. (Nanjing, China), which were housed on a 12 h light:12 h dark cycle at 22 ± 2 °C and had free access to food and water. The procedures of animal experiments were executed according to the NIH guide for the care and use of laboratory animals, and were approved by the Ethics Committee of the Nanjing Jinling Hospital. Primary mouse Sertoli cells were isolated from testis of 20-day old male ICR mice by a two-step enzyme digestion as previously described [19] with some modifications. Briefly, testes were decapsulated, digested with 0.25% trypsin (Gibco; Thermo Fisher Scientific, Inc.) at 37 °C in a rocking incubator for 4–6 min, and washed with PBS, so that interstitial cells can be removed. The isolated seminiferous tubules were then digested with 1 mg/ml collagenase I at 37 °C in a rocking incubator for 6–8 min to remove peritubular cells. A 200-mesh stainless steel filter was used to filter the homogenate. Following two times of PBS washing, cells were resuspended with DMEM/F12 supplemented with 10% FBS, seeded in dishes, and incubated in a humidified 34 °C, 5% CO_2_ incubator. After adherence for 4 h, Sertoli cells became attached to the bottoms of dishes, while germ cells were suspended in the medium. Thus the cells were washed with PBS twice to remove most germ cells, and a hypotonic solution (0.3 × HBSS) was used to treat the cells for 3 min, so that residual germ cells can be lysed and removed. The cells were then cultured in a humidified 34 °C, 5% CO_2_ incubator for 2–3 days before the experiments.

### Cell viability assay

To analyze cell viability, an MTT assay was conducted. Cells were seeded in 96-well plates at a density of 5 × 10^3^, and cultured overnight to allow for cell attachment. The cells were pre-treated with RSG (20 μM) for 2 h and then PA (0.2 or 0.4 mM) was applied. After PA treatment for 12 or 24 h, the cell culture medium in each well was discarded and replaced with 200 μl fresh DMEM/F12 without FBS, and 20 μl MTT stock was added. The plate was incubated at 37 °C for 4 h, then the medium was discarded and 150 μl DMSO was added to each well to dissolve the formazan, which was reduced from MTT by living cells. Finally, the absorbance was measured at 450 nm using a microplate reader (Bio-Rad Laboratories, Inc., Hercules, CA, USA). For dose- and time-dependent analysis of the effect of RSG on PA-induced cytotoxicity, TM4 cells were treated with RSG at the indicated concentrations 2 h before, simultaneously with, or 2 h after the beginning of PA treatment; all of the cells were treated with PA for 24 h except for the cells in the control group. The MTT assay was then performed to analyze cell viability. For cell morphological observations, TM4 cells were seeded in 6-well plates at a density of 15 × 10^4^ and cultured overnight for cell attachment before the indicated treatments were applied. Images were captured following cell treatments using a microscope (IX73; Olympus Corporation, Tokyo, Japan).

### ORO staining

Cells were stained with ORO to assess intracellular lipid accumulation. Briefly, the cells were seeded in 6-well plates at a density of 1 × 10^5^, and cultured overnight for cell attachment. After cell treatments, the cells were fixed with 4% paraformaldehyde and stained with the freshly diluted ORO working solution at room temperature for 1 h. After rinsing with 75% ethanol for 30 s and washing with PBS twice, the cells were counterstained with hematoxylin for 10 s. Observations were made and images were captured using a microscope (IX73; Olympus Corporation). For the quantification of lipid accumulation, the stained samples were washed with PBS and incubated at 37 °C to evaporate any remaining water. Then, 200 μl isopropanol was added to each well, and the plates were slowly agitated at room temperature for 10 min for the dissolution of ORO staining. Following this, the samples were transferred to a 96-well plate, and the absorbance was measured at 510 nm using a microplate reader (Bio-Rad Laboratories, Inc.).

### Analysis of PA endocytosis

To observe PA endocytosis, a fluorescently-labeled PA analogue BODIPY FL C16 was used. TM4 cells were pretreated with or without 20 μM RSG for 24 h, and then treated with 1 μM BODIPY FL C16 for 30 min. Once washed three times with PBS, the cells were fixed with 4% paraformaldehyde. Fluorescent images were captured using a fluorescence microscope (IX73; Olympus Corporation), and the mean fluorescence intensities were quantified using ImageJ version 1.32j software (National Institutes of Health, Bethesda, MD, USA).

### RNA extraction and reverse transcription-quantitative polymerase chain reaction (RT-qPCR)

The mRNA levels of carnitine palmitoyltransferase 1A (CPT1A), carnitine palmitoyltransferase 1B (CPT1B), diacylglycerol O-acyltransferase 1 (DGAT1) and diacylglycerol O-acyltransferase 2 (DGAT2) were quantified by RT-qPCR. Briefly, total RNA was extracted from cells using a Total RNA Isolation Kit (BEI-BEI Biotech, Zhengzhou, China). The PrimeScript RT Master Mix (Takara Bio, Inc., Otsu, Japan) was used for RT-PCR. qPCR was carried out using the AceQ qPCR SYBR Green Master Mix (Vazyme Biotech, Nanjing, China) following the manufacturer’s instruction. The samples were amplified and monitored using a Roche LightCycler 96 Real-time PCR system (Roche Diagnostics, Basel, Switzerland). The thermocycling conditions were: 95 °C for 10 min for initial denaturation, and 40 cycles of amplification consisting of 95 °C for 10 s and 60 °C for 30 s. The relative expression levels were calculated using the 2^-ΔΔCq^ method [20], and the gene 36B4 (also known as ribosomal protein lateral stalk subunit P0) was used as the internal control. The primers used were as follows: 36B4, forward 5’-GAAACTGCTGCCTCACATCCG-3′ and reverse 5’-GCTGGCACAGTGACCTCACACG-3′; CPT1A, forward 5’-CTCAGTGGGAGCGACTCTTCA-3′ and reverse 5’-GGCCTCTGTGGTACACGACAA-3′; CPT1B, forward 5’-TACAGCTTCCAAACGTCACTGCC-3′ and reverse 5’-CACCATGACTTGAGCACCAGG-3′; DGAT1, forward 5’-TCCGTCCAGGGTGGTAGTG-3′ and reverse 5’-TGAACAAAGAATCTTGCAGACGA-3′; DGAT2, forward 5’-GCGCTACTTCCGAGACTACTT-3′ and reverse 5’-GGGCCTTATGCCAGGAAACT-3′.

### Western blot analysis

The cells were lysed in Radioimmunoprecipitation Assay buffer for protein extraction [21]. Protein concentrations were analyzed using the Pierce™ BCA Protein Assay Kit (Thermo Fisher Scientific, Inc.), and 20 μg protein was loaded in each lane for gel electrophoresis. The following operations were done as previously described [21]. The primary antibodies used were as follows: Rabbit polyclonal CPT1A (1:2,000; cat. no. 15184–1-AP; ProteinTech Group, Inc., Chicago, IL, USA) and mouse monoclonal GAPDH (1:2,000; cat. no. KC-5G5; KangChen Biotech, Inc., Shanghai, China). The secondary antibodies used were as follows: Goat anti-rabbit IgG (H + L) secondary antibody, horseradish peroxidase (HRP)-conjugated (1:5,000; cat. no. 31460; Invitrogen; Thermo Fisher Scientific, Inc.) and goat anti-mouse IgG (H + L) secondary antibody, HRP-conjugated (1:5,000; cat. no. 31430; Invitrogen; Thermo Fisher Scientific, Inc.). The bands were visualized using enhanced chemiluminescence reagents (Promega Corporation, Madison, WI, USA), and images were captured using the Tanon-5200 Chemiluminescent Imaging System (Tanon Science and Technology, Co., Ltd., Shanghai, China). The relative protein expression levels were reflected by the intensities of the target bands, which were quantified using ImageJ version 1.32j software (National Institutes of Health).

### PPARγ RNAi

The mouse PPARγ-specific siRNA set (siPPARγ) and non-specific siRNA (scrambled siRNA, NC-siRNA) were designed and synthesized by Ribobio (Guangzhou, China). To knockdown the expression of PPARγ, the cells were transfected with NC-siRNA or siPPARγ 6 h in advance of indicated treatments. The transfection of siRNAs were performed using Lipofectamine 3000 reagent (cat. no. L3000015, Invitrogen; Thermo Fisher Scientific, Inc.) according to the manufacturer’s instructions.

### Statistical analysis

GraphPad Prism 5 software (GraphPad Software, Inc., La Jolla, CA, USA) was used for graph generation. The data are presented as the mean ± standard deviation. To compare the results between different groups, one-way analysis of variance followed by the Least Significant Difference (for equal variances) or the Games-Howell (for unequal variances) post hoc test were conducted using SPSS software version 17.0 (SPSS, Inc., Chicago, IL, USA). Differences were considered to be statistically significant when *P* < 0.05, and highly significant when *P* < 0.01.

## Results

### RSG ameliorates the decline in Sertoli cell viability induced by PA

**Fig. 1:**
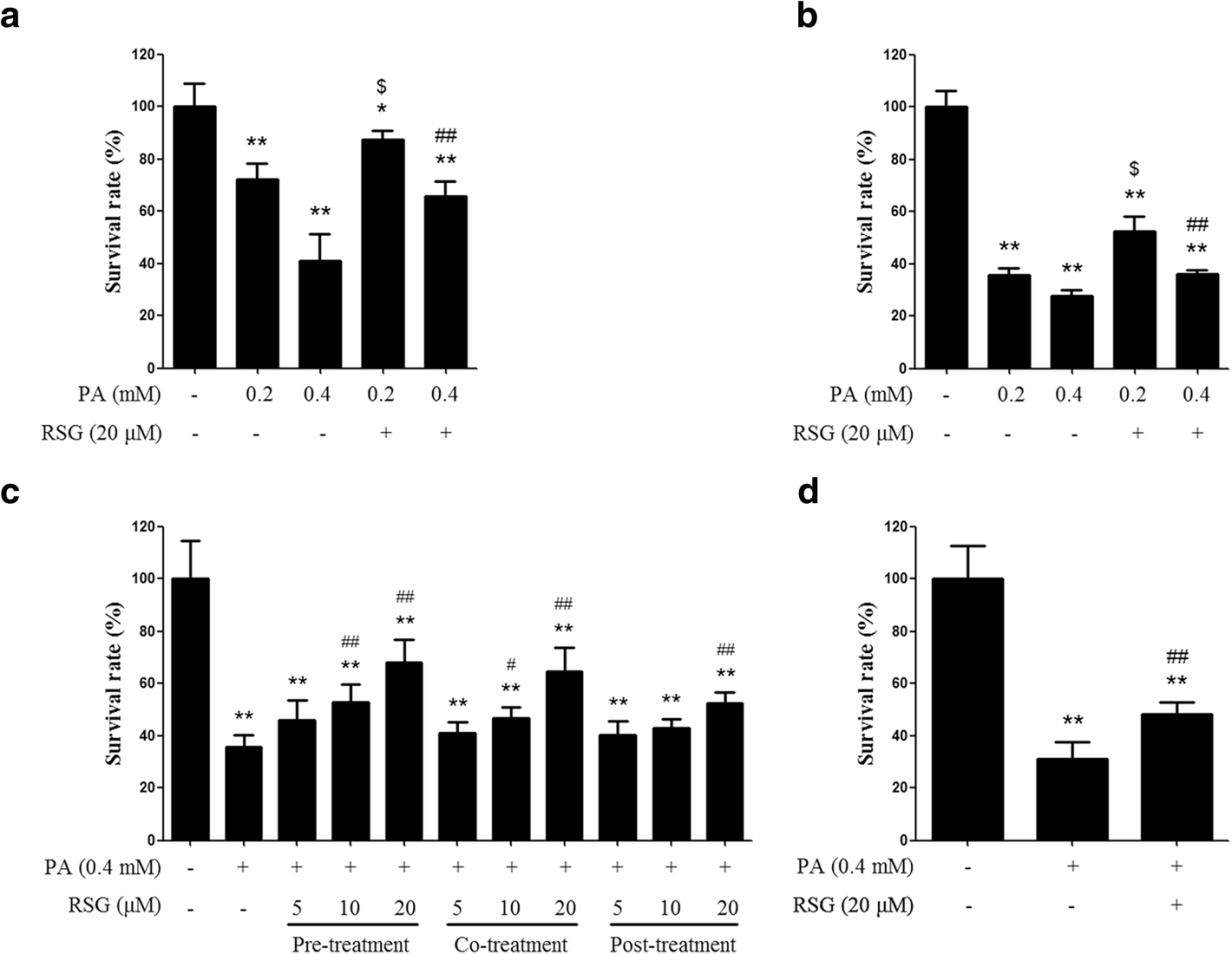
RSG ameliorates the PA-induced decrease in Sertoli cell viability. (**a** and **b**) An MTT assay was performed with TM4 cells treated with PA for (**a**) 12 h or (**b**) 24 h, with or without RSG pre-treatment. **c** Dose- and time-dependent analysis of the effect of RSG on PA-induced cytotoxicity (MTT assay). **d** An MTT assay was conducted in primary mouse sertoli cells treated with PA for 24 h with or without RSG pre-treatment. Data are presented as the mean ± standard deviation of three independently prepared samples, each with three measurements. ^*^P < 0.05 and ^**^P < 0.01 vs. control group; ^$^*P* < 0.05 vs. 0.2 mM-PA group; ^#^*P* < 0.05 and ^##^*P* < 0.01 vs. 0.4 mM-PA group. *RSG* rosiglitazone, *PA* palmitic acid, *MTT* 3-(4,5-dimethyl-2-thiazolyl)-2,5-diphenyl-2-H-tetrazolium bromide

To validate the toxicity of PA, TM4 Sertoli cells were treated with 0.2 or 0.4 mM PA for 12 or 24 h. The concentrations of PA used in the present study are in reference to the concentration of free fatty acids in circulation [22] and the concentration of PA commonly used in other studies [23, 24]. The MTT assay results revealed that both concentrations of PA decreased cell viability (Fig. 1a and b). However, the 20 μM RSG treatment, which was added 2 h prior to PA stimulation, significantly ameliorated this decline in cell viability, thereby indicating the potential protective effect of RSG in TM4 Sertoli cells (Fig. 1a and b). To verify the effect of RSG, a dose- and time-dependent experiment was executed. A total of 5, 10 or 20 μM RSG was added to cells 2 h prior to, simultaneously with, or 2 h after the addition of 0.4 mM PA, then cells were subsequently treated with PA for 24 h. Both the results of the MTT assay and the cell status observed by a microscope demonstrated that RSG exhibited protective effects in all these treatments (Fig. 1c and Additional file 1: Figure S1). Moreover, according to MTT results, the toxicity of PA, and the protective role of RSG, were also observed in primary mouse Sertoli cells (Fig. 1d).

### RSG alleviates PA-induced lipid accumulation in Sertoli cells

**Fig. 2:**
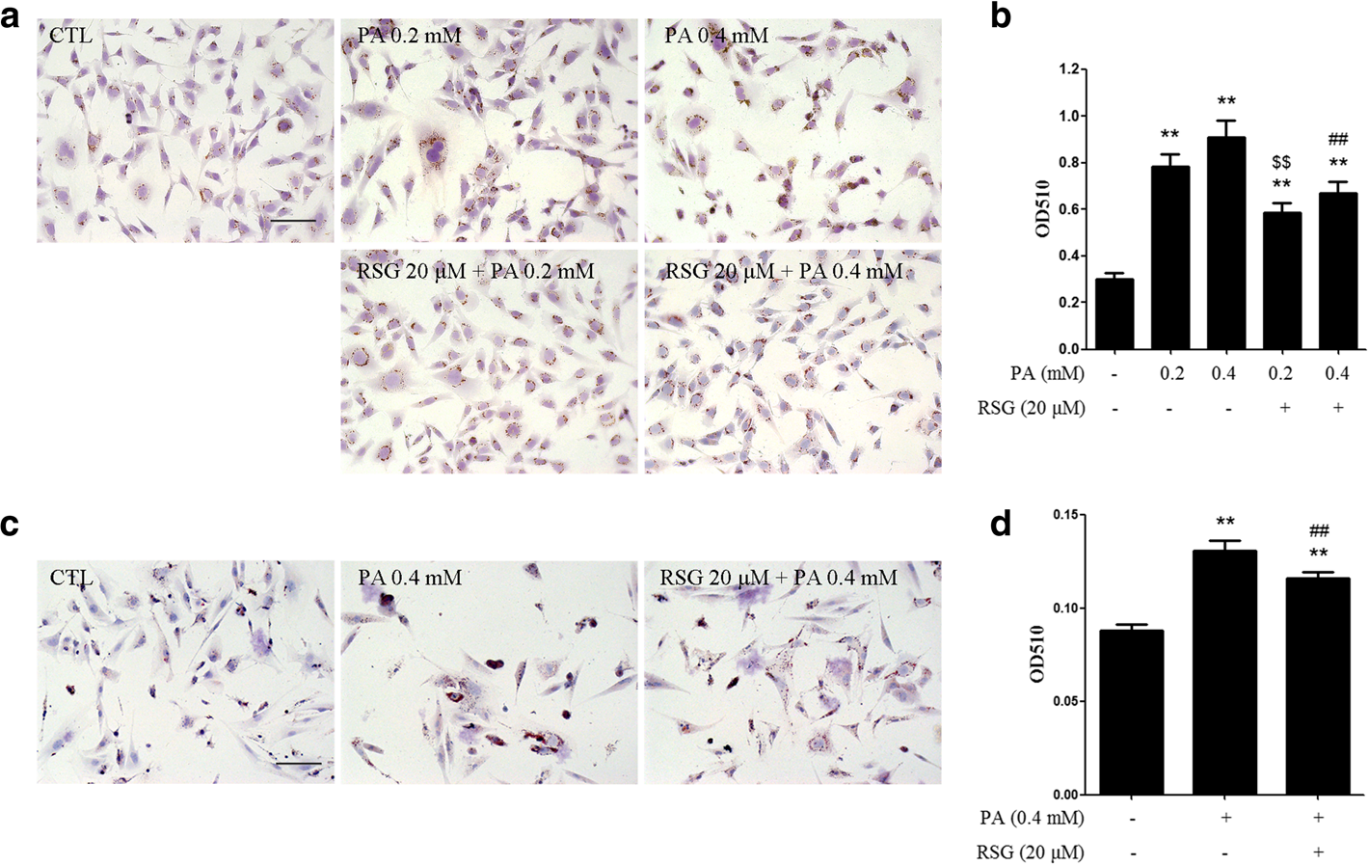
RSG alleviates PA-induced lipid accumulation in Sertoli cells. TM4 cells (**a** and **b**) and primary mouse Sertoli cells (**c** and **d**) were pre-treated with 20 μM RSG for 2 h, and then treated with 0.2 or 0.4 mM PA for 24 h. **a** and **b** ORO staining of TM4 cells (**a**) and quantification of neutral lipids (**b**). **c** and **d** ORO staining of primary mouse Sertoli cells (**c**) and quantification of neutral lipids (**d**). Data are presented as the mean ± standard deviation of three independently prepared samples, each with three measurements. Scale bar, 100 μm.^**^P < 0.01 vs. control group; ^$$^*P* < 0.01 vs. 0.2-mM PA group; ^##^*P* < 0.01 vs. 0.4 mM-PA group. *RSG* rosiglitazone, *PA* palmitic acid, *ORO* oil red O

To determine whether the protection from PA-induced cytotoxicity by RSG is due to reduced lipid accumulation in cells, ORO staining was performed to observe the neutral lipid droplets in cells. As was expected, treatment with PA significantly increased the levels of ORO staining in TM4 cells, indicating there was elevated lipid accumulation. When the cells were pretreated with RSG for 2 h, there was substantially less ORO staining of intracellular lipid droplets when compared with the cells treated with PA alone (Fig. 2a and b). Post-treatment with RSG showed a similar protective role (Additional file 1: Figure S2). In primary mouse Sertoli cells, pre-treatment with RSG also ameliorated PA-induced lipid accumulation (Fig. 2c and d). These results demonstrated that RSG may alleviate PA-induced lipid accumulation.

### RSG ameliorates PA-induced cytotoxicity through a PPARγ-dependent pathway

**Fig. 3:**
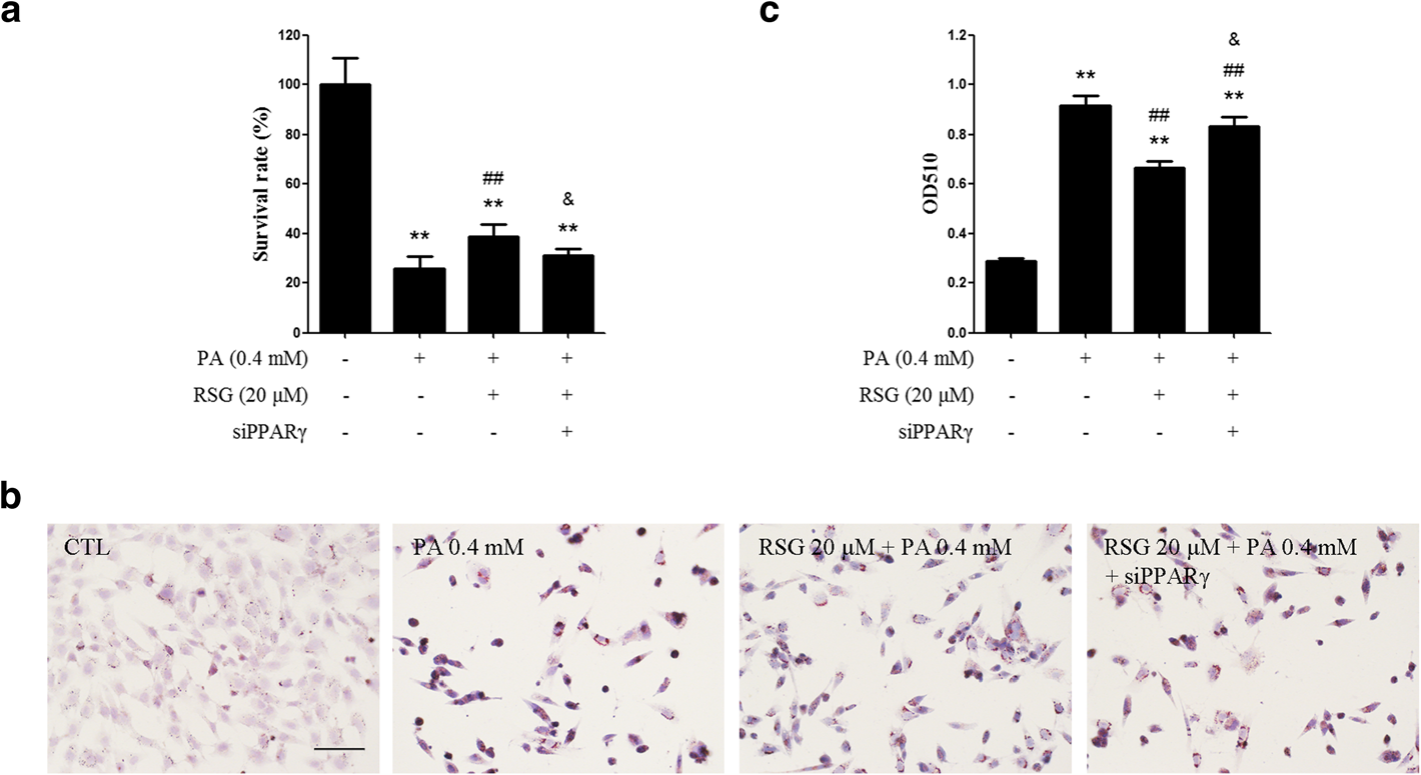
Knockdown of PPARγ alleviated the protective effects of RSG on PA-induced lipotoxicity in Sertoli cells. TM4 cells were transfected with NC-siRNA or siPPARγ. 6 h after transfection, cells were pretreated with (or without) RSG for 2 h, and then treated with PA for 24 h. **a** MTT assay of TM4 cells. **b** and **c** ORO staining of primary mouse Sertoli cells (**b**) and quantification of neutral lipids (**c**). Data are presented as the mean ± standard deviation of three independently prepared samples, each with three measurements. Scale bar, 100 μm. ^**^P < 0.01 vs. control group; ^##^*P* < 0.01 vs. 0.4 mM-PA group; ^&^*P* < 0.05 vs. 0.4 mM-PA + 20 μM-RSG group. *RSG* rosiglitazone, *PA* palmitic acid, *ORO* oil red O

RSG is a PPARγ agonist, so it may exert its protective effects through a PPARγ-dependent pathway. To investigate the involvement of PPARγ-dependent pathway, a set of PPARγ specific siRNAs was transfected into TM4 cells to knock down the expression of PPARγ. Both the MTT assay and ORO staining assay indicated that knocking down PPARγ expression substantially alleviated the protective effects of RSG on PA-induced lipotoxicity (Fig. 3). Therefore, it can be inferred that RSG protects Sertoli cells from PA-induced lipotoxicity through a PPARγ-dependent pathway.

### RSG does not suppress PA endocytosis

**Fig. 4:**
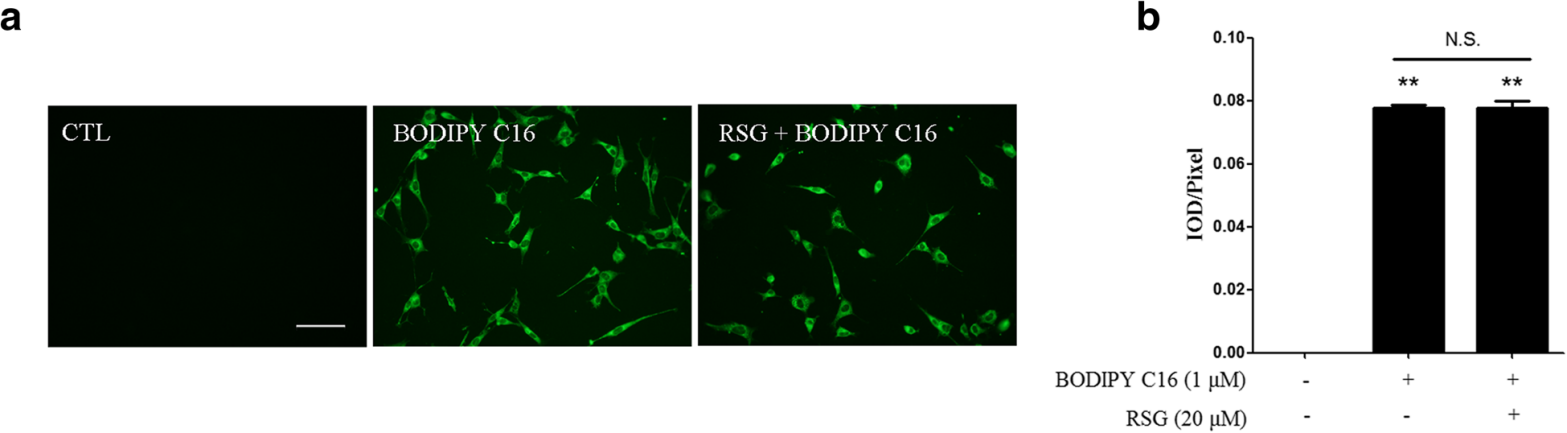
RSG did not affect PA endocytosis. TM4 cells were pretreated with or without 20 μM RSG for 24 h, and then treated with 1 μM BODIPY FL C16 for 30 min. **a** Fluorescent images were captured with a fluorescence microscope (scale bar, 100 μm), and (**b**) the mean fluorescence intensities were quantified. Data are presented as the mean ± standard deviation of three independent experiments. ^**^P < 0.01 vs. control group. N.S., not significant; RSG, rosiglitazone; PA, palmitic acid; BODIPY FL C16, 4,4-difluoro-5,7-dimethyl-4-bora-3a,4a-diaza-s-indacene-3-hexadecanoic acid

Decreased lipid accumulation may be due to a decrease in PA endocytosis, a decrease in lipid synthesis, or an increase in lipid catabolism. To evaluate whether RSG affects PA endocytosis, BODIPY FL C16, a PA analogue labeled with a fluorophore, was used to trace the uptake of PA. Notably, RSG pre-treatment did not suppress the endocytosis of PA (Fig. 4). Therefore, the inhibition of PA uptake does not explain the decreased levels of lipid accumulation following RSG treatment.

### RSG induces the expression of lipid catabolic genes

**Fig. 5:**
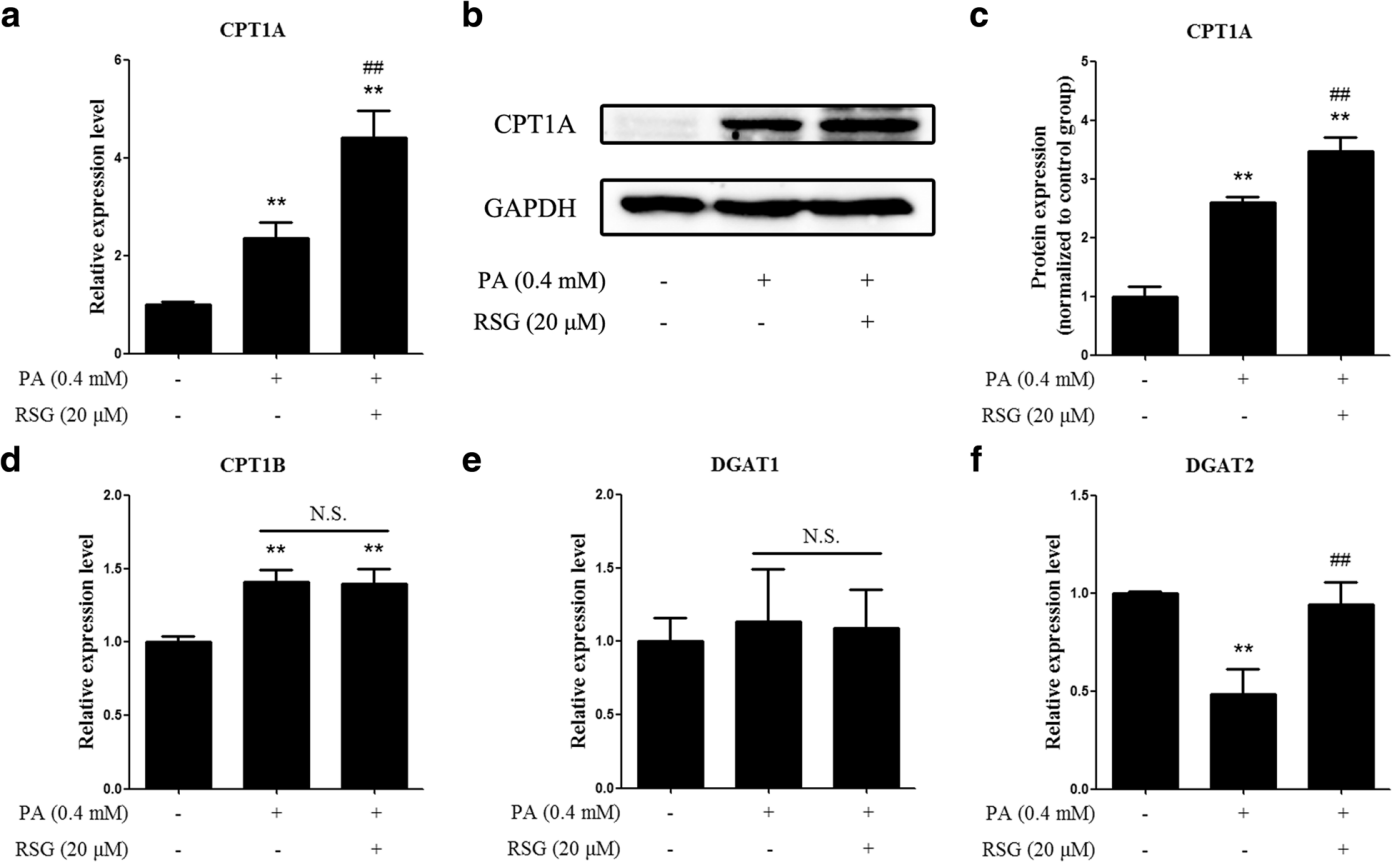
Effects of RSG on lipid metabolic genes. mRNA expression levels of (**a**) CPT1A, (**d**) CPT1B, (**e**) DGAT1 and (**f**) DGAT2 were assessed using the reverse transcription-quantitative polymerase chain reaction method. **b** Protein expression levels of CPT1A were validated using western blot analysis, and (**c**) quantified by densitometry. Data are presented as the mean ± standard deviation of three independent experiments. ^**^P < 0.01 vs. control group; ^##^*P* < 0.01 vs. 0.4 mM-PA group. N.S., not significant; RSG, rosiglitazone; PA, palmitic acid; CPT1A/1B, carnitine palmitoyltransferase 1A/1B; DGAT1/2, diacylglycerol O-acyltransferase 1/2

To clarify whether the RSG-induced alleviation of lipid accumulation was as a result of increased lipid catabolism or decreased lipid synthesis, the expression levels of key genes involved in both processes were measured. The results of RT-qPCR indicated that the expression of CPT1A, a gene that mediates fatty acid β-oxidation, was upregulated by PA and was further elevated by RSG (Fig. 5a). Similarly, western blot analysis of CPT1A also reflected these results (Fig. 5b and c). However, the expression of CPT1B, another gene associated with fatty acid oxidation, was also upregulated by PA, but was not affected by RSG (Fig. 5d). In addition, the upregulation of CPT1B (1.41-fold increase) by PA was not as marked as that of CPT1A (2.36-fold increase). DGAT1 and DGAT2, key enzymes that regulate the synthesis of triglyceride from fatty acids, were also detected by qPCR. The results demonstrated that DGAT1 levels were not significantly altered when compared among the three groups (Fig. 5e); however, DGAT2 mRNA expression was suppressed by PA, which was subsequently restored by RSG treatment (Fig. 5f). These results indicated that only the induction of CPT1A expression by RSG, which in turn led to increased fatty acid oxidation, may be able to explain how RSG decreases lipid accumulation.

### RSG ameliorates PA-induced cytotoxicity in HepG2 cells, but not in HUVECs

**Fig. 6:**
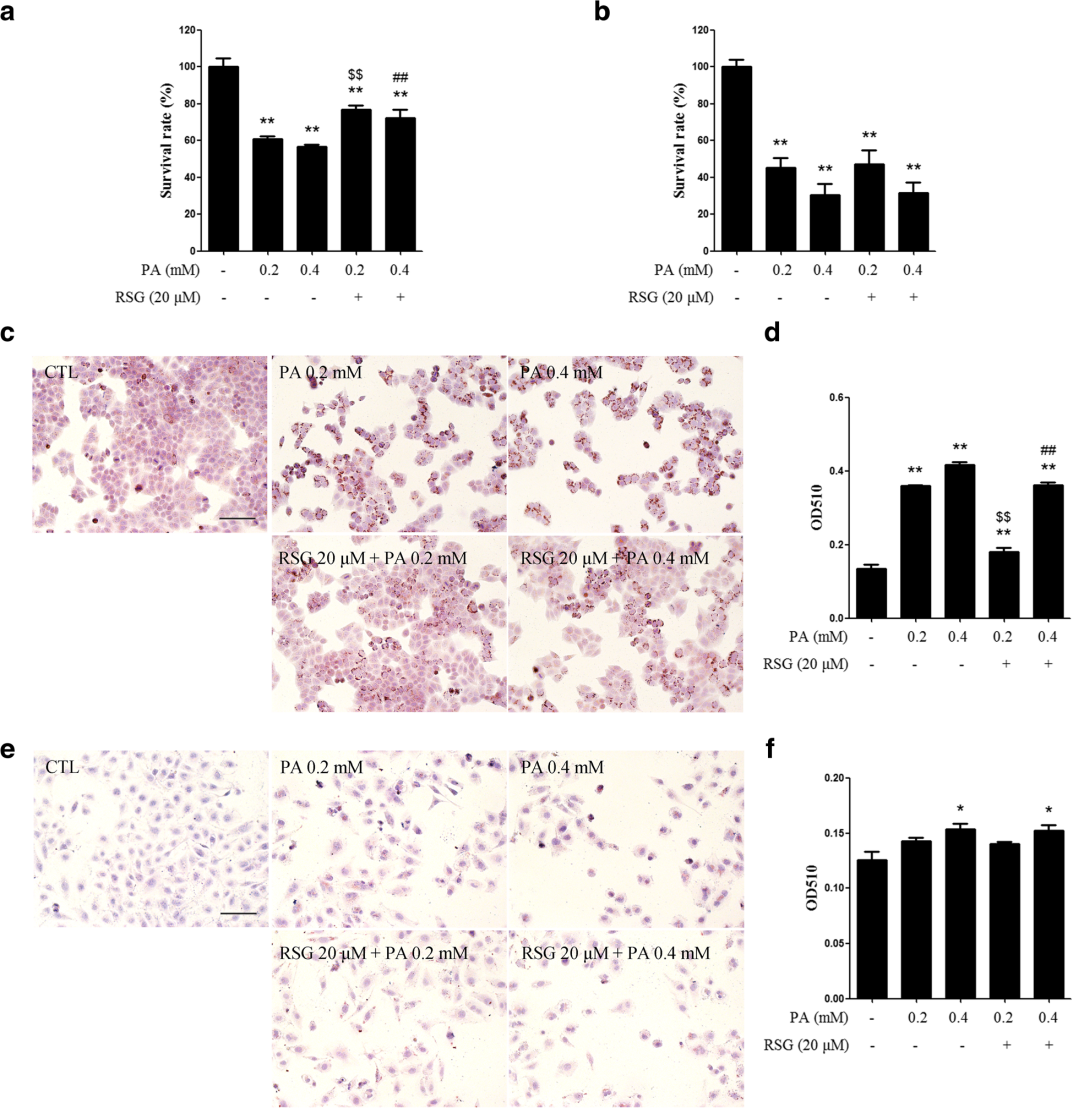
Effects of RSG on PA-induced cytotoxicity in HepG2 cells and HUVECs. **a** and **b** MTT assay of (**a**) HepG2 cells and (**b**) HUVECs treated with PA for 24 h with or without RSG pre-treatment. **c-f** ORO staining of (**c** and **d**) HepG2 cells and (**e** and **f**) HUVECs. Cells were pre-treated with 20 μM RSG for 2 h, and then treated with 0.2 or 0.4 mM PA for 24 h. **c** and **e** Scale bar, 100 μm. **d** and **f** Quantifications of neutral lipids in cells stained with ORO were also presented. Data are presented as the mean ± standard deviation of three independently prepared samples, each with three measurements. ^*^P < 0.05 and ^**^P < 0.01 vs. control group; ^$$^*P* < 0.01 vs. 0.2 mM-PA group; ^##^*P* < 0.01 vs. 0.4 mM-PA group. RSG, rosiglitazone; PA, palmitic acid; ORO, oil red O; HUVECs, human umbilical vein endothelial cells

To validate the specificity of the effect of RSG on different cell types, the present study selected a further two representative cell lines, HepG2 and HUVECs, which were analyzed by MTT assay and ORO staining. As previously reported, lipid metabolism in Sertoli cells is similar to that observed in hepatocytes [25, 26], thus, HepG2 was selected as a positive control. The results demonstrated that RSG significantly ameliorated the cytotoxicity and lipid accumulation induced by PA in HepG2 cells (Fig. 6a, c and d). In HUVECs, as the negative control, neither PA-induced cytotoxicity nor lipid accumulation was affected by RSG (Fig. 6b, e and f).

## Discussion

In non-adipose tissues, lipid accumulation in cells usually leads to cell dysfunction and apoptosis, which is known as lipotoxicity. Saturated fatty acids, such as PA and stearic acid, have been reported to be more toxic than other fatty acids [27]. In the present study, PA, which is the major saturated fatty acid in both plasma and spermatozoa, was selected as the representative saturated fatty acid. In our previous studies, the toxicity of PA on Sertoli cells has been demonstrated (unpublished data). In the present study, the results of the MTT assay also confirmed the toxicity of PA (Fig. 1). Moreover, lipid accumulation in cells caused by PA treatment was observed (Fig. 2), indicating there may be an imbalance of lipid metabolism.

RSG is an anti-diabetic drug and a PPARγ agonist. It has been reported to be beneficial to dyslipidemia, and exert protective effects in cells exposed to fatty acids, including PA [28, 29]. The present study investigated whether RSG protected Sertoli cells from PA-induced toxicity. According to the MTT results, RSG significantly elevated cell survival rates, which were decreased by PA (Fig. 1). In addition, the ORO staining assay indicated that RSG ameliorated the lipid accumulation induced by PA (Fig. 2). Furthermore, knocking down PPARγ by its specific siRNAs significantly abolished the protective effects of RSG (Fig. 3). These results demonstrated that RSG may serve a cytoprotective role in Sertoli cells exposed to PA, and PPARγ play a part in the actions of RSG in Sertoli cells. It is worth mentioning that post-treatment with 20 μM RSG also showed a considerable effect on PA treated Sertoli cells (Fig. 1c and Additional file 1: Figure S2), indicating that RSG has the potential not only in the prevention but also in the therapy of Sertoli cell dysfunction coupled with dyslipidemia.

There are several possible mechanisms by which lipid accumulation may be attenuated in cells induced by fatty acids: i) prevent the endocytosis of fatty acids; ii) inhibit the synthesis of triglyceride; and iii) promote the clearance of fatty acids by β-oxidation [30]. To elucidate the mechanism by which RSG alleviates PA-induced lipid accumulation in Sertoli cells, the present study evaluated the uptake of PA after RSG treatment, and detected the expression of genes involved in triglyceride synthesis and β-oxidation.

PA-induced lipid accumulation is accompanied by an increase in fatty acid uptake, as demonstrated by the increased translocation of fatty acid transporters, such as cluster of differentiation-36 (CD36), after PA treatment [31]. Therefore, inhibiting the cellular uptake of fatty acids has an effect on limiting lipotoxicity [32]. To investigate the cellular uptake of PA, a fluorescent PA analogue, BODIPY FL C16, was added to the culture medium of TM4 Sertoli cells. BODIPY FL C16 taken up by cells would exhibit fluorescence inside the cells, which can be observed using a fluorescence microscope [33]. According to the results of the present study, the endocytosis of PA was not significantly different when comparing cells with and without RSG pre-treatment (Fig. 4). Therefore, it could be concluded that RSG does not affect the uptake of PA into Sertoli cells, and thus, the protective effect of RSG on PA-induced lipid accumulation in Sertoli cells may be due to a change in the balance between fatty acid β-oxidation and triglyceride synthesis.

As previously reported, redirection of PA metabolism towards oxidation exerts a protective role in cells against PA-induced toxicity [34]. CPT1s are enzymes that mediate the binding of long-chain fatty acids to carnitine and promote their transport across mitochondrial membranes. Therefore, CPT1s catalyze the rate-limiting step of fatty acid β-oxidation [35]. CPT1A and CPT1B are two isoforms of CPT1s. Upregulation of the expressions of CPT1A and CPT1B leads to a decrease in lipid accumulation [36]. Thus, the expression levels of CPT1A and CPT1B were determined in the present study. The results demonstrated that CPT1A expression increased following PA stimulation, and further upregulation of CPT1A was observed after RSG treatment (Fig. 5a-c). However, although the expression of CPT1B was induced by PA, the extent was not as marked as that observed with CPT1A. Moreover, the expression of CPT1B was not elevated further by RSG treatment (Fig. 5d), which indicated that CPT1B may be not involved in the protective effect of RSG. Taken together, these results suggested that RSG may induce fatty acid oxidation by upregulating CPT1A expression, which in turn may protect Sertoli cells from PA-induced cytotoxicity.

DGATs are enzymes that catalyze the formation of triglycerides from diglycerides, which is the final step in triglyceride synthesis. In mammals, DGAT1 and DGAT2 are the two isoforms of DGATs [37]. Inhibition of either DGAT1 or DGAT2 has been considered to be an attractive target for the treatment of dyslipidemia [38]. However, the effects of DGATs on lipotoxicity remain unclear. For example, in cardiomyocytes, an acute overexpression of DGAT1 serves a protective role; however, prolonged overexpression of DGAT1 causes excessive lipid accumulation and leads to cell dysfunction [39]. Therefore, the effects of DGATs should be considered, depending on the circumstances. In the present study, it was revealed that DGAT1 expression did not change following PA stimulation or RSG pre-treatment (Fig. 5e). On the other hand, DGAT2 expression was markedly decreased by PA treatment, and RSG pretreatment restored its expression (Fig. 5f). As DGAT2 is an enzyme involved in lipid synthesis, its upregulation by RSG may not explain the induced decrease in lipid accumulation. However, the acute DGAT2 overexpression induced by RSG may be a protective mechanism; nevertheless, it may not compromise the catabolic effect of CPT1A on PA.

Notably, these results regarding the expression of CPT1s and DGATs were quite similar to those of Joung Hoon Ahn et al. [30], who reported the protective effects of oleic acid against PA-induced pancreatic AR42J cell apoptosis. According to their results, DGAT1 expression remained unchanged, while DGAT2 expression was inhibited by PA and upregulated by oleic acid. In addition, CPT1 expression was also induced by PA and further elevated by oleic acid. Both RSG and oleic acid are PPARγ activators [40], thus, it is possible that activation of PPARγ may serve a protective role in PA-induced cell damage. As previously reported, PPARγ is also involved in the regulation of both CPT1s and DGATs [41, 42]. Therefore, it follows that PPARγ may participate in the regulation of fatty acid metabolism, and activation of PPARγ may be an effective treatment for Sertoli cell dysfunction induced by saturated fatty acids.

The effects of RSG on lipid metabolism are dissimilar in different types of tissues [43], possibly due to their different metabolic patterns. As previously reported, lipid metabolism in Sertoli cells is similar to that observed in hepatocytes [25, 26]. Also, the existence of lipid droplets is a feature of Sertoli cells [44], indicating that excessive lipid can be stored as lipid droplets in Sertoli cells. Similarly, hepatocytes also store lipid in large quantities, which is different from other cell types such as epithelial cells [45]. Therefore, the hepatocytic cell line HepG2 and the epithelial cell line HUVEC were selected in the present study as positive and negative controls, respectively. In HepG2 cells, RSG significantly rescued cell viability, which was decreased by PA stimulation (Fig. 6a). In addition, the lipid accumulation induced by PA was also alleviated by RSG (Fig. 6c and d). By contrast, cell viability was not affected by RSG in HUVECs (Fig. 6b). Moreover, the accumulation of lipid droplets in HUVECs after PA treatment was not as marked as that observed in HepG2 and TM4 cells, and RSG did not decrease this lipid accumulation (Fig. 6e and f). Therefore, the effect of RSG in protecting cells from lipotoxicity may be specific to Sertoli cells and hepatocytes, and not to other cell types that do not store excess lipid in large quantities. These results indicated that the pattern of lipid metabolism is similar in Sertoli cells and hepatocytes, and so the treatment strategies used for improving liver steatosis may have potential as a therapy for Sertoli cell dyslipidemia.

## Conclusions

The results of the present study demonstrated that RSG ameliorated the toxicity caused by PA in Sertoli cells by inducing CPT1A expression to promote fatty acid oxidation. According to these findings, RSG, and possibly other PPARγ agonists, offer potential in protecting Sertoli cells from saturated fatty acid-induced cytotoxicity. Moreover, TM4 cells appear suitable for analyzing mechanisms involved in Sertoli cell steatosis and its reversal. These results provide novel insights into the development of therapeutic methods for the treatment of Sertoli cell dysfunction coupled with dyslipidemia; however, the curative effect of RSG requires confirmation both in vivo and in clinical trials.

## Additional file

Additional file 1: **Figure S1.** Dose- and time-dependent analysis of the effect of RSG on PA-induced cytotoxicity (cell morphological observations). **Figure S2.** Post-treatment with RSG alleviates PA-induced lipid accumulation in TM4 cells. (PDF 382 kb)

## Abbreviations

36B4/RPLP0: Ribosomal protein lateral stalk subunit P0
BODIPY FL C16: 4,4-difluoro-5,7-dimethyl-4-bora-3a,4a-diaza-s-indacene-3-hexadecanoic acid
BSA: Bovine serum albumin
CPT1A/1B: Carnitine palmitoyltransferase 1A/1B
DGAT1/2: Diacylglycerol O-acyltransferase 1/2
DMEM/F12: Dulbecco’s modified Eagle’s medium/Ham’s nutrient mixture F12
DMSO: Dimethyl sulfoxide
FBS: Fetal bovine serum
MTT: 3-(4,5-dimethyl-2-thiazolyl)-2,5-diphenyl-2-H-tetrazolium bromide
ORO: Oil red O
PA: Palmitic acid
PBS: Phosphate buffer saline
PPARγ: Peroxisome proliferator-activated receptor-γ
RSG: Rosiglitazone
RT-qPCR: Reverse transcription-quantitative polymerase chain reaction
SD: Standard deviation
