# Supplementary material for: Rosiglitazone ameliorates palmitic acid-induced cytotoxicity in TM4 Sertoli cells
Source: Reprod Biol Endocrinol. 2018 Oct 17;16:98. doi: 10.1186/s12958-018-0416-0 (PMC6192158; doi:10.1186/s12958-018-0416-0)
Supplement: Supplementary file 1 — Figure S1. Dose- and time-dependent analysis of the effect of RSG on PA-induced cytotoxicity (cell morphological observations). Figure S2. Post-treatment with RSG alleviates PA-induced lipid accumulation in TM4 cells. (PDF 382 kb) [file 12958_2018_416_MOESM1_ESM.pdf]

Additional file 1

Rosiglitazone ameliorates palmitic acid-induced cytotoxicity in TM4 Sertoli cells

Xie Ge [1†], Peng Pan [1†], Jun Jing [1], Xuechun Hu [1], Li Chen [1], Xuhua Qiu [1], Rujun Ma [1], Kadiliya Jueraitetibaike [1], Xuan Huang [1] and Bing Yao [1*]

[1] Center of Reproductive Medicine, Nanjing Jinling Hospital, Clinical School of Medical College, Nanjing University, Nanjing 210002, Jiangsu, China.

[†] These authors contributed equally to this work.

[*] Author to whom correspondence should be addressed; E-Mail: yaobing@nju.edu.cn; Tel.: +86-25-8086-0174; Fax: +86-25-8086-0174.

Supplementary Results

Supplementary Figures

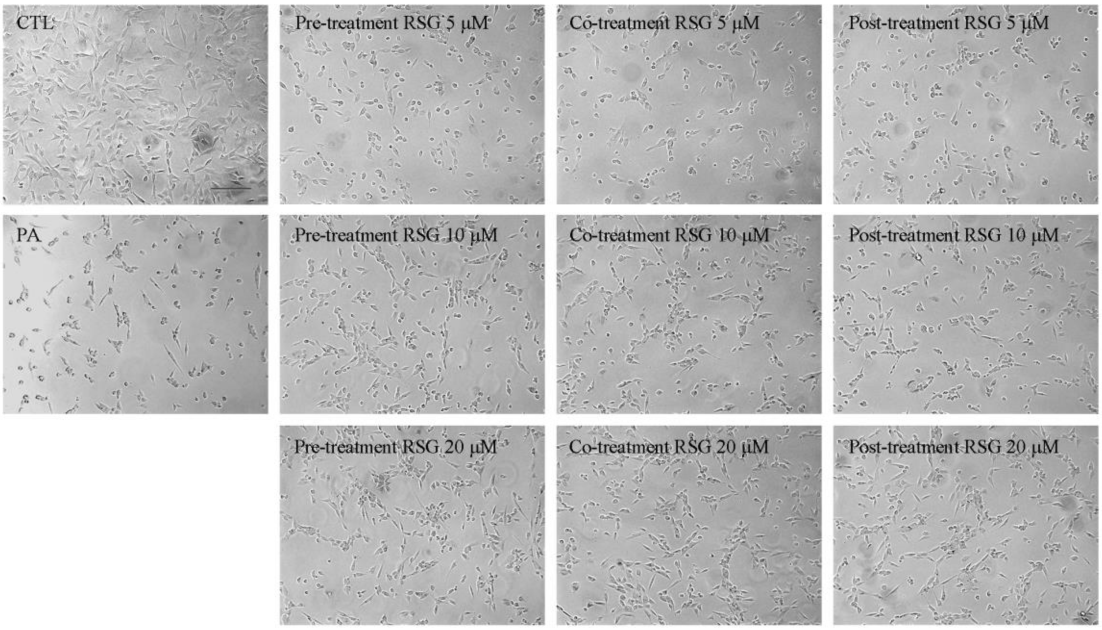

Figure S1. Dose- and time-dependent analysis of the effect of RSG on PA-induced cytotoxicity (cell morphological observations). TM4 cells were treated with RSG at the indicated concentrations 2 h before (Pre-treatment), simultaneously with (Co-treatment), or 2 h after (Post-treatment) the beginning of PA treatment; all of the cells were treated with PA for 24 h except for the cells in the control group. Images were captured following cell treatments using a microscope. Scale bar, 200 μm. RSG, rosiglitazone; PA, palmitic acid.

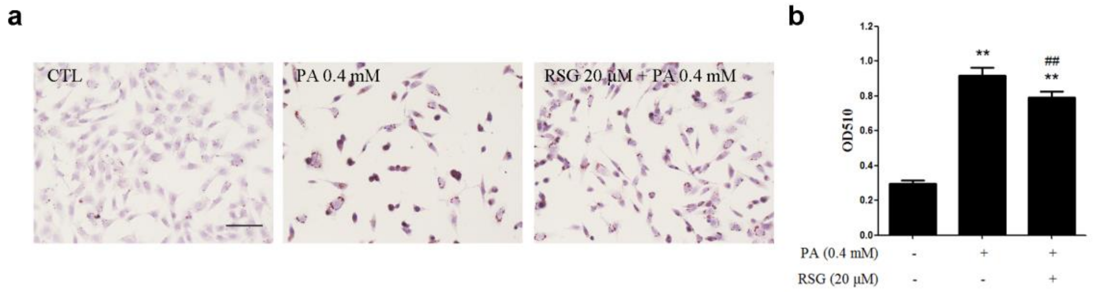

Figure S2. Post-treatment with RSG alleviates PA-induced lipid accumulation in TM4 cells. (**a**) ORO staining of TM4 cells. Cells were treated with 0.4 mM PA for 2 h, and then treated with both 0.4 mM PA and 20 μM RSG for 24 h. Scale bar, 100 μm. (**b**) Quantification of neutral lipids in cells stained with ORO. Data are presented as the mean ± standard deviation of three independently prepared samples, each with three measurements. **$P<0.01$ vs. control group; ##$P<0.01$ vs. 0.4 mM-PA group. RSG, rosiglitazone; PA, palmitic acid; ORO, oil red O.
